# Congenital Diarrhea: A Case Report on Challenges in Both Prenatal and Postnatal Diagnosis

**DOI:** 10.1155/crog/9987632

**Published:** 2026-08-25

**Authors:** H. Heinrich, E. Pajkrt, M. M. Tabbers, E. van Leeuwen

**Affiliations:** ^1^ Department of Obstetrics and Gynaecology, Amsterdam UMC, University of Amsterdam, Amsterdam, Netherlands, uva.nl; ^2^ Amsterdam Reproduction and Development Research Institute, Amsterdam, Netherlands; ^3^ Department of Pediatrics/Pediatric Gastroenterology and Nutrition, Emma Children′s Hospital, Amsterdam UMC, University of Amsterdam, Amsterdam, Netherlands, uva.nl; ^4^ Amsterdam Gastroenterology Endocrinology Metabolism Research Institutes, Amsterdam, Netherlands

## Abstract

Prenatal recognition of congenital diarrhea and subsequent diagnosis can facilitate neonatal monitoring and prompt initiation of lifesaving postnatal treatment. We describe two cases with a congenital diarrheal disorder (CDD), highlighting the prenatal course and the diagnostic process. In cases of fetal dilated bowel loops, particularly with a characteristic honeycomb appearance, prenatal genetic testing for CDDs should be considered. After birth, distinguishing diarrhea from urine may be challenging in neonates with suspected congenital diarrhea. Therefore, urinary catheterization or a urine collection bag is recommended to ensure accurate assessment.

## 1. Case Report

We report two cases of congenital diarrhea with prenatal dilated bowel loops to evaluate the prenatal and postnatal diagnostic process and possible pitfalls.

### 1.1. Case 1

A male fetus was referred to the fetal medicine unit at 21 weeks of gestation following the second trimester anomaly scan due to suspected dilation of the fetal bowel. Prenatal screening (noninvasive prenatal testing [NIPT]) did not reveal aneuploidies. Dilated fetal bowel was confirmed, with a suspected small bowel obstruction. No other structural anomalies were suspected. There was no family history of gastroenterological diseases. Based on initial sonographic findings, parents waived genetic testing but opted for screening for congenital infections and cystic fibrosis carrier screening, which yielded negative results.

**Figure 1:**
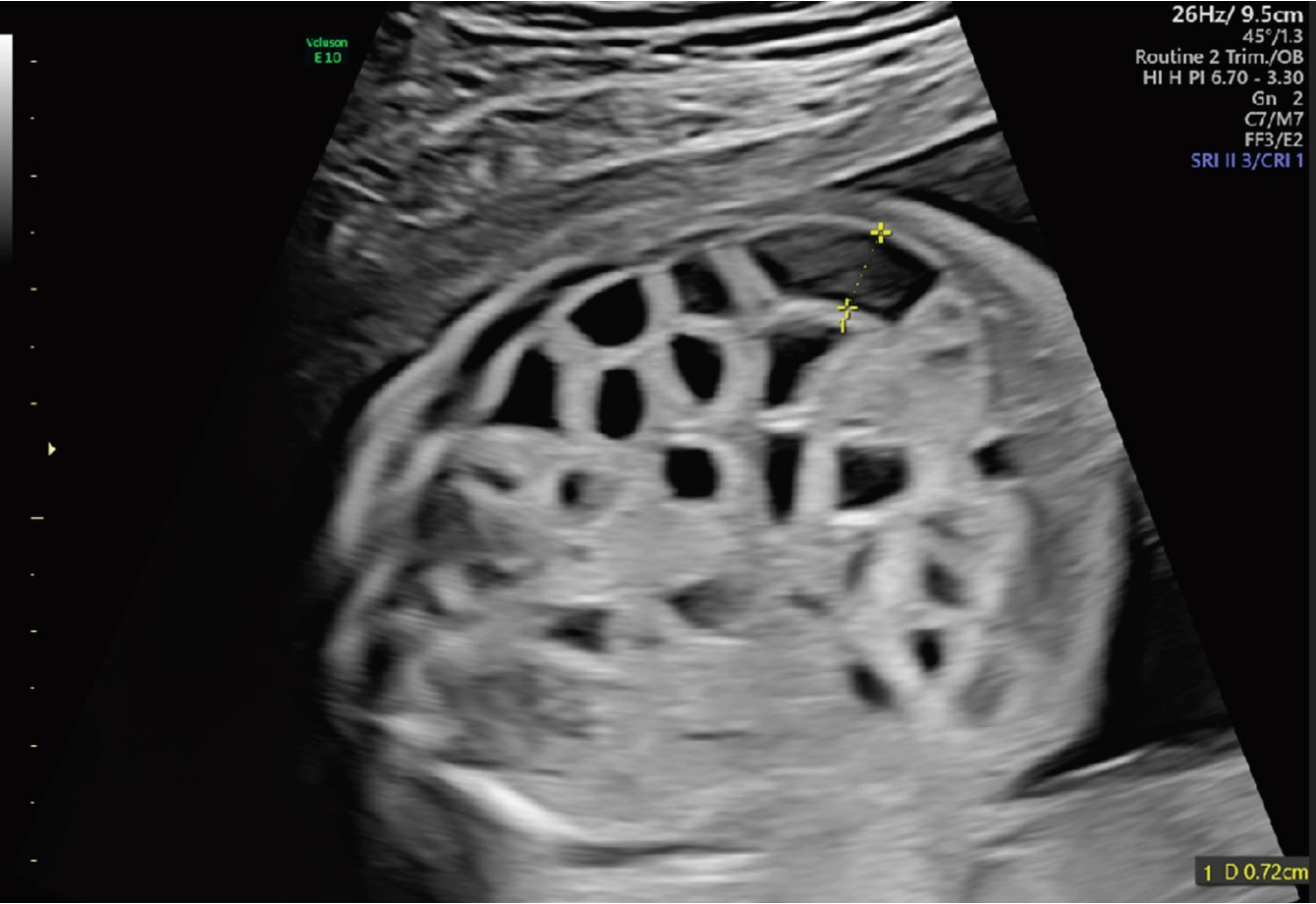
Case 1, dilated bowel loops at 23 weeks of gestation in patient with a SLC9A3 mutation (ultrasound image).

**Figure 2:**
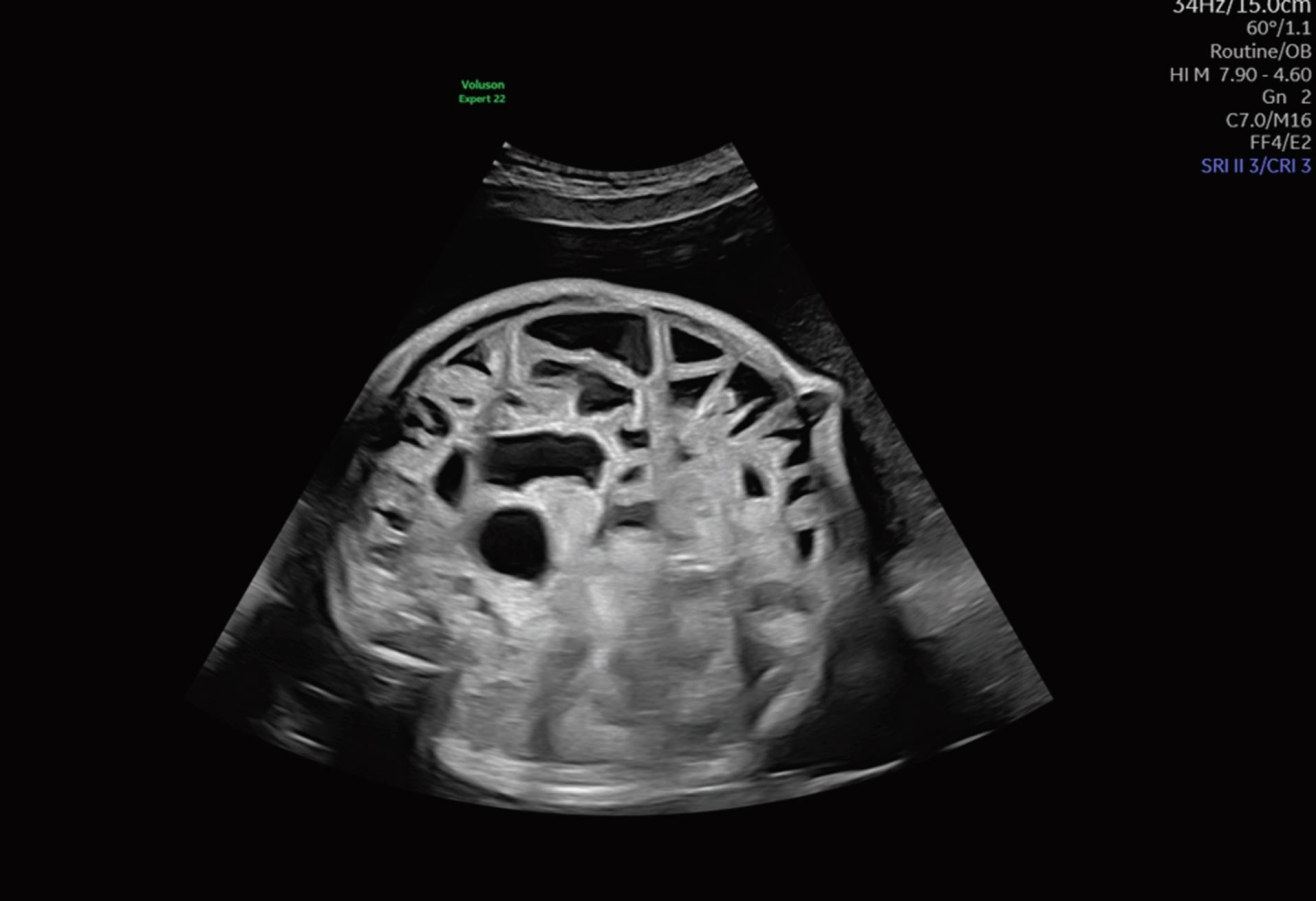
Case 1, dilated bowel loops at 35 weeks of gestation with “honeycomb aspect” and polyhydramnios in patient with a SLC9A3 mutation (ultrasound image).

The pregnancy was complicated by polyhydramnios from 29 weeks of gestation. During follow‐up ultrasound examination, due to the typical honeycomb appearance of the dilated bowel loops, the diagnosis of small bowel obstruction was abandoned and the suspicion of congenital diarrhea arose (Figures 1 and 2). Due to rapid increase of amniotic fluid and maternal discomfort, amnioreduction was performed at 32 and 34 weeks of gestation and subsequent genetic testing was performed. The color of the amniotic fluid was a light green. QF‐PCR and array analysis revealed no abnormality. However, prior to receiving the result of the whole exome sequencing (WES), premature rupture of the membranes resulted in delivery at a gestational age of 36 weeks. The baby was admitted to the ICU because of the prenatal diagnosis of congenital diarrhea for careful monitoring of the vital signs and stool output. A consultation was done by pediatric gastroenterologists. Abdominal x‐ray first day postpartum did not reveal a bowel obstruction and enteral feed was initiated and well tolerated. Because of the antenatal findings and the need for amnioreduction, the baby was not dismissed home and was transferred to general pediatric care department with the intention to do close monitoring of the stool output.

During postnatal follow‐up, in the first week after birth, the content of the wet diapers was interpreted as normal urine. However, the infant developed a metabolic acidosis on day 9 after birth. The metabolic acidosis was corrected with sodium chloride infusion, glucose infusion, and nil by mouth. Eleven days postpartum WES of the amniotic fluid identified a compound heterozygous mutation of the SLC9A3 gene. One missense mutation was found to be heterozygous in the mother, but the second variant (a frameshift mutation) was not detected in either parent. This suggests that the frameshift mutation is a de novo mutation. Total parental nutrition (TPN) was initiated from the day of the diagnosis onwards and the metabolic acidosis was corrected. Parents were trained to perform TPN in the home setting, after which the infant was discharged from the hospital after 44 days.

### 1.2. Case 2

At 36+3 weeks of gestation, parents were referred to the fetal medicine unit from a secondary hospital after progressive dilated bowel loops were detected on ultrasound examination at 34 and 35 weeks of gestation. The pregnancy had been uneventful, including normal NIPT results, no structural anomalies on the second trimester anomaly scan, apart from gestational diabetes without medication from 24 weeks of gestation onwards. Other than ulcerative colitis in the father, there was no family history of gastroenterological diseases. Due to the gestational diabetes and a history of fetal demise in a previous pregnancy, prenatal ultrasound monitoring was intensified.

**Figure 3:**
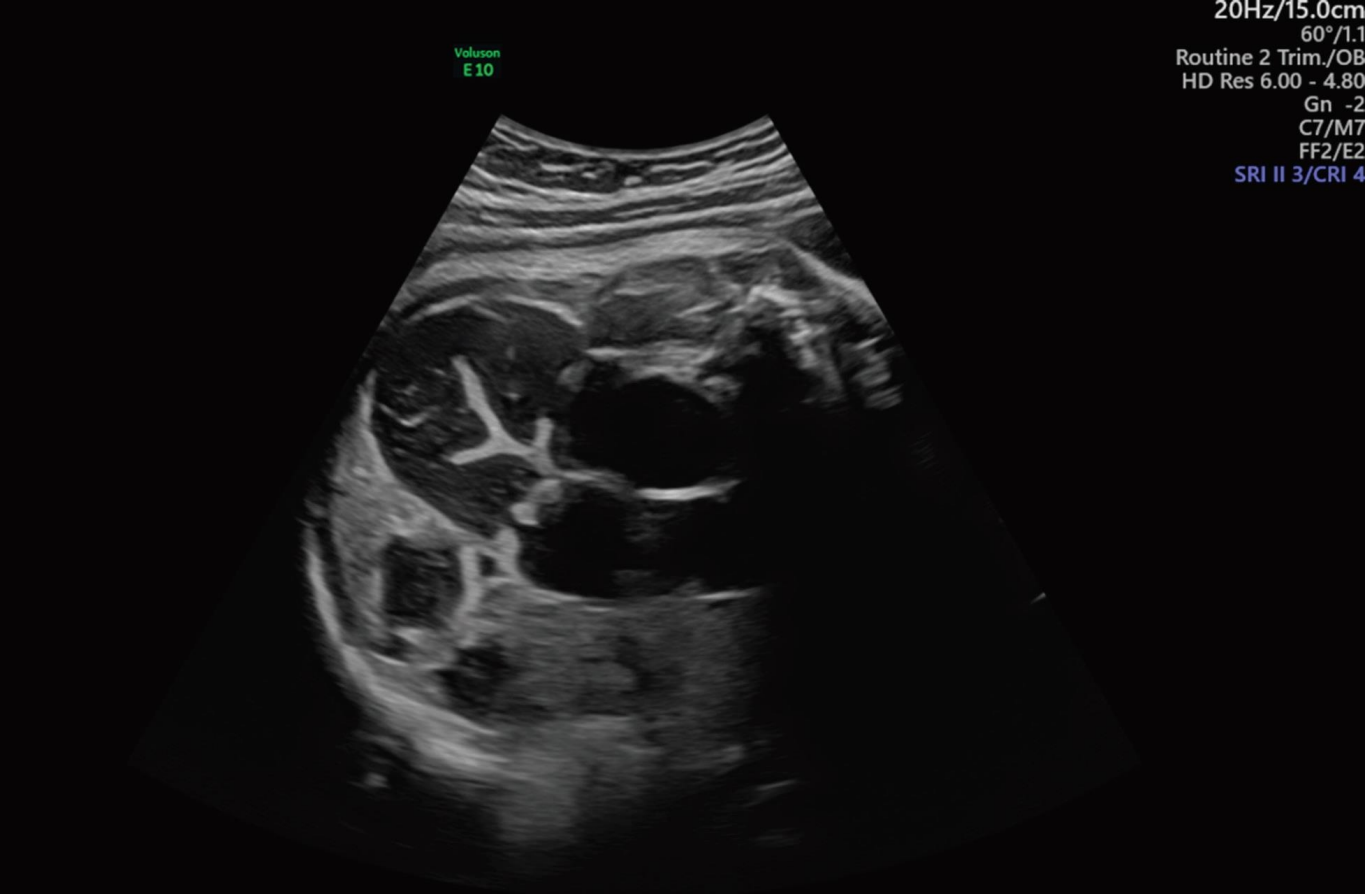
Case 2, dilated bowel loops at 36 weeks of gestation in patient with a MYO5B mutation (ultrasound image).

During the advanced ultrasound examination, dilatation of the large bowel up until 21 mm was observed, though amniotic fluid measurements were normal (Figure 3). The differential diagnoses, including bowel obstruction, cystic fibrosis, Morbus Hirschsprung, or other underlying causes, were discussed with future parents. Given the advanced gestation, invasive diagnostics were not pursued. Daily monitoring by cardiotocography was initiated, and delivery from 37 weeks onwards was discussed.

However, parents returned later the same day (36+3weeks) because of spontaneous onset of labor. A primary cesarean section was performed because of breech position of the fetus. The male neonate was admitted to the pediatric ward for observation and abdominal x‐ray 6 hours postpartum showed some gas in the small intestine, but none in the rectum. A tube could be inserted in the rectum without any signs of obstruction and meconium at the end of the probe. Therefore, partial oral feeding was initiated, which was well tolerated, supplemented by enteral feeding. Abdominal x‐ray 12‐h postpartum revealed dilated bowel loops, but since the neonate exhibited no other symptoms (distended abdomen, spontaneous stool) expectative management was continued. Three days postpartum, the neonate was transferred to a nonacademic hospital and discharged home 5 days postpartum.

However, 9 days postpartum, the neonate was readmitted because of significant weight loss and possible increase in stool frequency. Upon physical examination a dehydrated neonate was observed, and blood examination revealed a severe (hyperchloremic) metabolic acidosis. The acidosis was corrected with sodium bicarbonate and sodium chloride infusion, as well as oral rehydration solutions. Further testing was conducted to rule out renal tubular acidosis, which could not be confirmed. Ultrasound evaluation of the kidneys also revealed no structural abnormalities or obstructions. Although metabolic disease testing was initiated, this was considered unlikely. Because of the suspicion of an enteropathy including microvillus inclusion disease (MVID), a gastroduodenoscopy was performed 5 days later. The definitive diagnosis of MVID was based on histopathology results of the small bowel. Although the metabolic acidosis was compensated for within 24 h, the neonate developed diarrhea, initially mistaken for urine. The acidosis was solely attributed to enteral bicarbonate loss. TPN was initiated from the day of the diagnosis onwards and after 28 days the neonate was discharged from the hospital. DNA diagnostic testing revealed two deletions in the MYO5B gene, with both parents identified as carriers.

## 2. Discussion

Congenital diarrheal disorders (CDDs) encompass a variety of enteropathies with onset early in life [1]. Postpartum diagnosis is often known to have a severe delay, in which time the neonate has suffered from severe dehydration and electrolyte disturbances [2, 3]. Neonates with congenital diarrhea present with watery stools, which can be mistaken for urine [4]. Therefore, correct prenatal diagnosis can decrease the time to initiation of treatment and prevent unnecessary surgical interventions [5, 6].

As CDDs encompass a heterogeneous group of conditions, several classifications for CDDs have been proposed [1, 7]. Terrin et al. proposed a classification of CDDs into four subgroups [1]. These include defects in the digestion, absorption, and transport of nutrients and electrolytes, such as mutations affecting solute carriers (e.g., SLC9A3 in our first case), defects in enterocyte differentiation and polarization, including MVID as described in our second case, defects in enteroendocrine cell differentiation, and abnormalities in the regulation of the intestinal immune response. Nevertheless, the genetic basis of some CDDs remains unresolved.

During the prenatal phase, recognizing congenital diarrhea can be challenging due to its similarities to other gastrointestinal abnormalities such as small bowel obstructions, which also commonly presents with polyhydramnios. However, the suspected mechanism for polyhydramnios in small bowel obstructions is the interference of absorption of amniotic fluid distal to the obstruction, whereas in congenital diarrhea, this is thought to be the consequence of the excessive loss of watery diarrhea [8, 9]. Subsequently, the dilated bowel in congenital diarrhea is a consequence of the accumulation of fluid in the gastrointestinal tract. Prenatally, the “honeycomb” image has been described in cases with congenital diarrhea, in which multiple dilations of the intestines are observed as adjacent anechoic spaces in the fetal abdomen [10, 11]. This honeycomb appearance was identified in our first case, but this was less pronounced in the second case. In addition, some studies suggest that increased peristalsis could be found in cases with small bowel obstruction whereas normal peristalsis is more often observed in congenital diarrhea [5]. Prenatal diagnosis can be complemented by amniocentesis for karyotyping and genetic testing, specifically by WES or targeted DNA. In addition, differentiation through MRI or biochemical testing of the amniotic fluid has been described to further distinguish congenital diarrhea from other causes of polyhydramnios and dilated bowel loops [4, 8]. It should also be considered that cases may be missed due to the absence of a routine third‐trimester ultrasound in the Dutch prenatal screening program. Since gastrointestinal abnormalities often become more apparent in the third trimester, prenatal diagnosis may not occur, leading to a subsequent delay in postnatal diagnosis [12].

In our first case, parents waived genetic testing upon the initial suspicion of a small bowel obstruction, as they had prior received normal NIPT results and screening for congenital infections and cystic fibrosis carrier screening yielded negative results. However, necessity for amnioreduction occurred in the third trimester, and additional genetic testing was initiated, of which the WES results unfortunately were not available at the time of spontaneous preterm delivery. An available appropriate diagnosis of the congenital sodium diarrhea prior to delivery might have facilitated postnatal initiation of treatment even sooner. In the second case, diagnosis of the dilated bowel loops occurred at 36+3 weeks of gestation, resulting in insufficient time for appropriate prenatal diagnostics. Due to the lack of genetic testing prenatally and no clinical signs or abnormalities, the neonate was discharged from the hospital and presented days later with a potentially life‐threatening metabolic acidosis.

Postnatal detection of congenital diarrhea is of the utmost importance to prevent severe dehydration. Distinguishing watery diarrhea from urine can be challenging and diarrhea can be mistaken for polyuria and can lead to the suspicion of Bartter syndrome for instance [4]. We encountered this difficulty in our cases, where the differentiation between urine and watery diarrhea proved to be challenging. In cases with a suspected congenital diarrhea, differentiation between urine and feces could be improved by placing a urine collection bag after birth. Treatment after birth consists of parenteral nutrition, with suppletion of possible deficient electrolytes, in order to survive and to maintain body growth [13]. The course of the diseases is very heterogeneous. The introduction of enteral feeding can increase stool output and thereby enhance electrolyte instability [14]. However, classic CSD improves over time and cases have been described in which patients with congenital diarrhea could be weaned completely from parenteral nutrition in childhood [7, 15, 16].

In conclusion, accurately diagnosing CDDs in the absence of conclusive genetic results remains challenging. In case of dilated bowel loops, the possibility of a CDD should be considered and discussed with future parents. If parents choose to undergo invasive genetic testing prenatally, analyses for CDDs should be considered, particularly in the presence of the typical honeycomb appearance of the dilated bowel loops. After birth, the use of a urine collection bag or catheter should be considered in cases with prenatally identified dilated bowel loops to correctly distinguish between urine and feces, thereby reducing the risk of delayed diagnosis.

## Author Contributions

H.H.: conceptualization, visualization, writing—original draft. E.P.: writing—review and editing. M.M.T.: writing—review and editing. E.L.: conceptualization, writing—review and editing, and supervision.

## Funding

No funding was received for this manuscript.

## Consent

The patients have provided informed consent.

## Conflicts of Interest

The authors declare no conflicts of interest.

## Data Availability

The data that support the findings of this study are available from the corresponding author upon reasonable request.
